# Motion guided Spatiotemporal Sparsity for high quality 4D-CBCT reconstruction

**DOI:** 10.1038/s41598-017-17668-5

**Published:** 2017-12-12

**Authors:** Yang Liu, Xi Tao, Jianhua Ma, Zhaoying Bian, Dong Zeng, Qianjin Feng, Wufan Chen, Hua Zhang

**Affiliations:** Guangdong Provincial Key Laboratory of Medical Image Processing, Southern Medical University, Guangzhou, Guangdong, 510515 China

## Abstract

Conventional cone-beam computed tomography is often deteriorated by respiratory motion blur, which negatively affects target delineation. On the other side, the four dimensional cone-beam computed tomography (4D-CBCT) can be considered to describe tumor and organ motion. But for current on-board CBCT imaging system, the slow rotation speed limits the projection number at each phase, and the associated reconstructions are contaminated by noise and streak artifacts using the conventional algorithm. To address the problem, we propose a novel framework to reconstruct 4D-CBCT from the under-sampled measurements—Motion guided Spatiotemporal Sparsity (MgSS). In this algorithm, we try to divide the CBCT images at each phase into cubes (3D blocks) and track the cubes with estimated motion field vectors through phase, then apply regional spatiotemporal sparsity on the tracked cubes. Specifically, we recast the tracked cubes into four-dimensional matrix, and use the higher order singular value decomposition (HOSVD) technique to analyze the regional spatiotemporal sparsity. Subsequently, the blocky spatiotemporal sparsity is incorporated into a cost function for the image reconstruction. The phantom simulation and real patient data are used to evaluate this algorithm. Results show that the MgSS algorithm achieved improved 4D-CBCT image quality with less noise and artifacts compared to the conventional algorithms.

## Introduction

Three-dimensional cone-beam computed tomography (3D-CBCT) has been widely used in image guided radiation therapy (IGRT)^1–3^. It can provide the volumetric information for tumor localization in IGRT. But for thoracic and upper abdominal regions, the 3D-CBCT image is often deteriorated by motion blur^4^. To address the problem, four-dimensional CBCT (4D-CBCT) imaging that incorporating temporal (phase) information on the basis of 3D-CBCT was proposed^5–7^. Compared to 3D-CBCT imaging, 4D-CBCT can provide the patient-specific respiratory motion information and multiple three-dimensional volumes to represent the different status in the breathing cycle^8–10^. For 4D-CBCT imaging, the respiratory signal is recorded or estimated and motion contained cone-beam projections are usually sorted into 8–10 subsets according to the respiratory signal. However, the gantry rotation speed and frame rate of the flat-panel imager limit the total number of cone-beam projections (usually 600~800), which result in relatively fewer projections in each respiratory phase. Consequently, the reconstructed CBCT images by using the conventional Feldkamp–Davis–Kress (FDK) algorithm^11^ suffer from significant artifacts and noise^12,13^. In addition, the randomness of breathing would lead to the cone-beam projections bunched into several clusters, and the bunched sampling scheme will aggravate the noises and artifacts level in the reconstructed images^14^.

To address this problem, various strategies have been proposed to improve the image quality of 4D-CBCT^15^. On one side, increasing the number of angular sampling, multiple-gantry rotation and slow-gantry rotation schemes were structured^7,13^. But these schemes will prolong the time of data acquisition and increase the risk of motion embracing. On the other side, lots of reconstruction algorithms^16–18^ have been proposed to improve the quality of 4D-CBCT image, such like the total variation minimization based algorithms^15,19,20^, the McKinnon–Bates (MKB) algorithm^21,22^, the prior image constrained compressed sensing (PICCS) algorithm^23^ and the auto-adaptive phase correlation (AAPC) algorithm^24^. As shown in the study by Frank Bergnera *et al*., the reconstruction algorithms using an iterative scheme can remarkably reduce the 4D-CBCT specific artifacts^25^. Nevertheless, algorithms that use the full data set, at least for initialization, such as MKB and PICCS algorithm, are only a trade-off and achieve sub-optimal temporal resolution, of which the residual motion could still be found in the reconstructed 4D-CBCT images^26^. On the other hand, algorithms that only use the projections assigned to the current phase to reconstruct the final image can fully achieve the temporal resolution, such as the total variation minimization based algorithm. For these algorithm, when the projection number is very low or there exist small size and low-contrast objects, tiny structure may be usually erased with the piecewise smooth constraint.

In recent years, the image restoration or denoising algorithms based on block processing have shown increasing vitality. The earliest concept of ‘patch’ was proposed in Haralick’s study on textural features for image classification^27^. In 1980, JS Lee pioneeringly used this concept for image enhancement^28^. Latterly, algorithms such like the dictionary learning based image reconstruction/restoration approaches^29^, the nonlocal means filter^30^ and the BM3D algorithm^31^, *et al*.^32–34^, have been successfully developed. Local patches often experience much less distortion than the global image and therefore it becomes easier to define the similarity between local patches. Low-rank and sparsity can be better reflected in patch based processing.

In this work, we propose to reconstruct 4D-CBCT volumes from the subset projections of current phase and incorporate the image domain phase-correlated information into the iterative procedure. Motived by the success of block based image restoration, we propose a Motion guided Spatiotemporal Sparsity (MgSS) to formulate the regularization for 4D-CBCT reconstruction. In this scheme, CBCT images of different phases are divided into small cubes (three-dimensional blocks) and the cubes are tracked with estimated motion vector fields through time (phase). After then, regional spatiotemporal sparsity is applied on the tracked cubes. Specifically, we recast the tracked cubes into four-dimensional matrix, and use the higher order singular value decomposition (HOSVD)^35^ technique to analyze the regional spatiotemporal sparsity. Finally, a cost function is formulated with embedding the block based spatiotemporal sparsity. One simple but effective optimization algorithm was used for the cost function solution.

This paper is structured as follows: In the method part, we first present the flow chart of the proposed MgSS scheme and then give detailed introduction of each step. After this, we formulate the reconstruction framework that incorporates the MgSS scheme. In the experiment part, we exhibit the results of the NCAT phantom simulation data, 4DCT based simulation data and real patient data by using FDK, SART-TV and the MgSS algorithms. Lastly, in the discussion part, we simply discuss the superiority and limitation of the proposed algorithm.

## Methods

### Flow chart of the proposed MgSS algorithm

In this work, the proposed Motion guided Spatiotemporal Sparsity (MgSS) applying to 4D-CBCT reconstruction comprises the following steps;

1. Estimate the motion maps for each voxel between adjacent phases of the 4D-CBCT images.
2. Divide the CBCT sequence images into cubes (3D blocks), and track the cubes through time using the three-dimensional motion maps obtained in step 1.
3. Stack the tracked cubes and apply regional spatiotemporal sparsity on the tracked cubes by framing the 4D-CBCT reconstruction problem to be a constrained optimization problem, and then optimize the problem to get the reconstructions.

In the following sections, we will introduce these steps in detail.

### Motion Maps Estimation

In this study, we are not devoted to develop new algorithm to achieve the three-dimensional motion vector fields (3D-MVFs) between CBCT images of adjacent phases, but rather we use the Real-Time Image-based Tracker (RTIT)^36^ toolbox to estimate the three-dimensional motion maps. The RTIT toolbox is an available open source with implementation of the optical flow based registration algorithms^37,38^. Assume that we obtain the initial/current 4D-CBCT estimation, with the RTIT, we can achieve the pixel based motion vector fields consisting of the changes in space coordinates that describe the distribution of the apparent motion velocities of intensity patterns in the sequences of images.

### Cubes Tracking with MVFs

**Figure 1:**
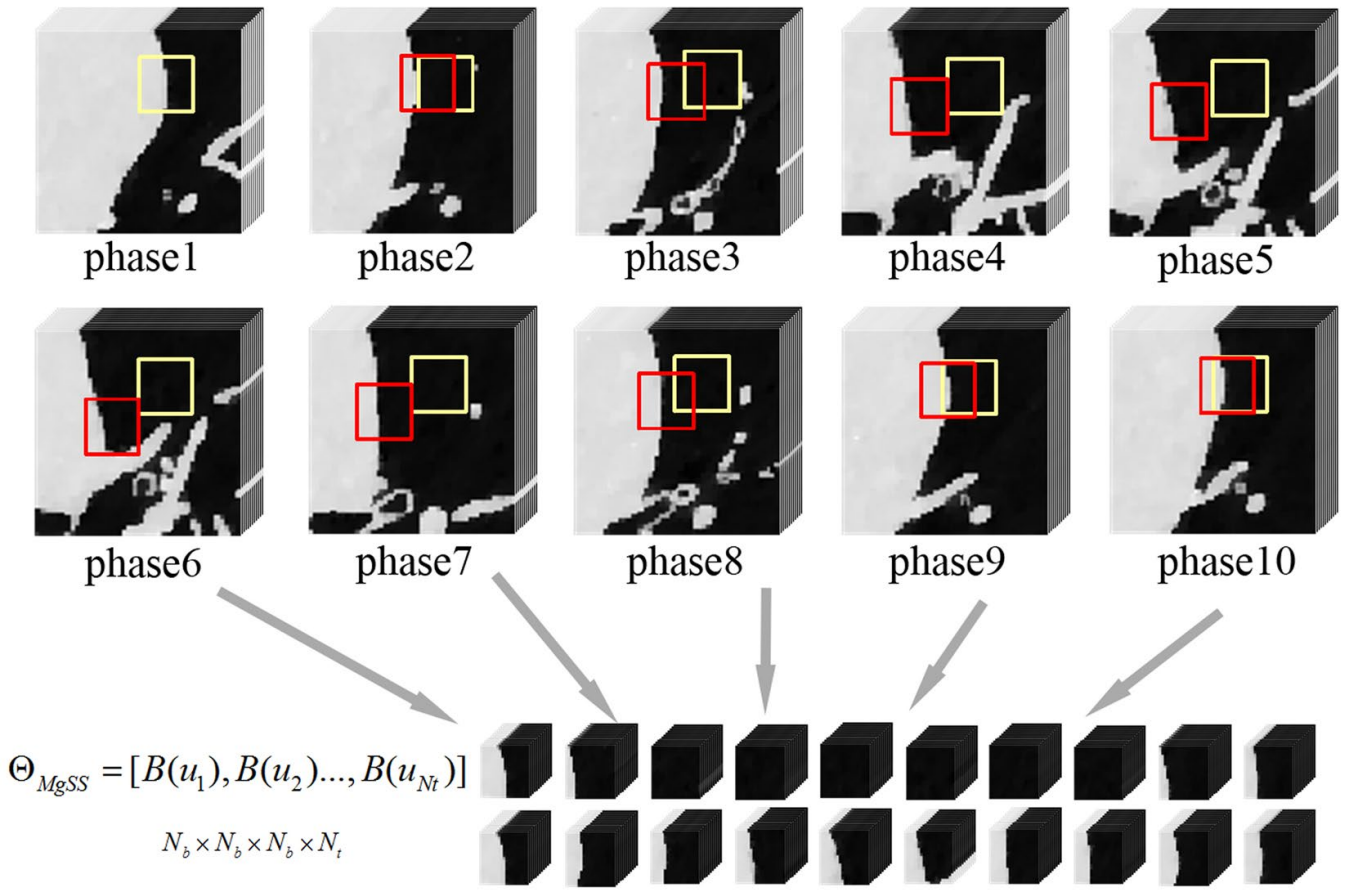
An example of blocks tracked (red squares) and not tracked (yellow squares) through all the frames.

For the task of cubes tracking, we define the voxel space-time position as $u=(x,\,y,\,z,\,t)$, where *x, y, z* and *t* represent the spatial position of the voxel in the CBCT volumetric image and the temporal phase index, respectively. In MgSS, we fetch the cubes (3D blocks) from the images of the first phase and use the 3D-MVFs to track structurally similar cubes in the other phases. Specifically, the first phase image was initiated with highly overlapping cubes. In the extreme case, all the voxels in the first phase image can be defined as the central voxel of one cube, but this may result in huge computation burden. Thus in this study, rather than sliding by one voxel to every next, we use a step of N_step_ = 2 voxels to define the cubes. For the motion tracking, considering one cube $B({u}_{1})\in {{X}_{1}}^{{N}_{b}\times {N}_{b}\times {N}_{b}}$, here ${u}_{1}=({x}_{1},\,{y}_{1},\,{{\rm{z}}}_{1},\,{t}_{1})$ indicates the central pixel position of the cubes with spatial position to be $({x}_{1},\,{y}_{1},\,{{\rm{z}}}_{1})$ and temporal phase index to be *t*_1_. By using Δ*u* to denote the displacement of the central voxel in the cube between *t*_1_ and *t*_2_ phases, we can use *u*_1_ + Δ*u* to track the center pixel location for cube in the *t*_2_ phase. Considering *u*_1_ + Δ*u* might be non-integral, the next block center voxel was taken as *u*_2_ = {*u*_1_ + Δ*u*_2_}, where “{}” is a rounding operation. Extracting the voxels centered from *u*_2_, we can constitute a new cube *B*(*u*_2_) with the same block size. Following this schedule, *B*(*u*_*n*_) can be tracked with *u*_*n*_= {*u*_1_ + Δ*u*_*n*_} using the above block-center-tracking method. A block cluster can be ulteriorly constructed: ${{\rm{\Theta }}}_{MgSS}=[B({u}_{1}),B({u}_{2}),\mathrm{...},B({u}_{Nt})]$. Here the cluster Θ_*MgSS*_ is a four dimensional matrix with the size of ${N}_{b}\times {N}_{b}\times {N}_{b}\times {N}_{t}$. Based on the fact that not all the chest is moving during the CBCT scan, the tracked cubes for the static parts should be noisy blocks with same anatomy structures. As shown in Fig. 1, compared to the blocks of the same spatial position in the original phases, the tracked blocks exhibit more mutual similarity.

### Regional Spatiotemporal Sparsity

The technology about matrix rank sparsity has been successfully investigated in the field of dynamic image reconstruction, such as the k-t SLR method^39^. In some previous studies^40^, matrix rank sparsity was often used to dispose the entire image. But the recent studies, such like Low-dimensional-structure self-learning and thresholding (LOST)^41^ and compartment-based k-t principal component analysis^42^ have indicated that the spatiotemporal sparsity and reconstruction quality could be further promoted by separating the entire image into blocks. Under the patch-based processing theory, decomposition of the tracked regions of dynamic datasets using the singular value decomposition (SVD) algorithm has been reported^43^. In Chen’s work, blocks in the clusters were vectorized and each cluster Θ was first rearranged into a 2-D matrix $\tilde{{\rm{\Theta }}}\in {{\mathbb{C}}}^{{N}_{s}\times {N}_{t}}({N}_{s}={N}_{b}\times {N}_{b})$. The SVD technique was then adopted to decompose the cluster: ${\tilde{{\rm{\Theta }}}}_{svd}=U{S}_{svd}{V}^{\ast }$. If the cluster is truly spatiotemporal sparsity, a least number of significant values will be found in the singular matrix *S*_*svd*_. For the standard SVD, the image blocks are manually vectorized and then the image structural properties in the spatial domain are ignored. To address the problem, the higher order singular value decomposition (HOSVD), which is able to directly decompose dynamic datasets into a multidimensional singular matrix rather than unfolding the blocks into column vectors, has been reported^35^. Also, in this work, we use the HOSVD to decompose the clusters due to its naturality and flexibility. By using the HOSVD technique, a four-dimensional tensor Θ can be decomposed as:

1 $${\rm{\Theta }}={S}_{hosvd}{\times }_{1}{U}^{(1)}{\times }_{2}{U}^{(2)}{\times }_{3}{U}^{(3)}{\times }_{4}{U}^{(4)}$$

where *U* ^(1)^, *U* ^(2)^, *U* ^(3)^, *U* ^(4)^ are orthogonal matrices that contain the orthonormal vectors spanning the column space of the matrix Θ. Here, the symbol ×_n_ stands for the n-th mode tensor product. The core tensor *S*_*hosvd*_ is not necessarily diagonal matrix, which implies that each dimension of the tensor can have a different rank. Generally, noise and artifacts can be attenuated by approximating the rank of the core tensor. However, the computation of the best rank approximation requires an iterative alternated least-square (ALS) algorithm and is quite time consuming^44^. Moreover, the approximation obtained from simple truncation has been proved to be in most cases quite similar to the optimal approximation^45^. Base on this, in our study, we apply a soft-thresholding strategy on the HOSVD coefficients to reduce noise and artifacts. The thresholding of the rank coefficients can be represented as follows:

2 $${\hat{S}}_{hosvd}={H}_{\tau }({S}_{hosvd})$$

where *H*_*τ*_ denotes the soft-thresholding operator with threshold *τ*. Then a new tensor can be synthesized by the inverse HOSVD transformation with truncated coefficients ${\hat{S}}_{hosvd}$ and the orthogonal matrices *U* ^(1)^, *U* ^(2)^, *U* ^(3)^, *U* ^(4)^:

3 $${{\rm{\Theta }}}_{hosvd}^{\ast }={\hat{S}}_{hosvd}{\times }_{1}{U}^{(1)}{\times }_{2}{U}^{(2)}{\times }_{3}{U}^{(3)}{\times }_{4}{U}^{(4)}$$

The above operation is repeated for each reference cube, thus provides multiple estimates at the same coordinate. For this reason, the final estimates are aggregated by weighted averaging all the obtained block-wise estimates that overlapped at each voxel.

### MgSS based 4D-CBCT reconstruction

To incorporate the motion tracking induced blocky spatiotemporal sparsity into the CBCT reconstruction framework, in this section, we formulate the following minimization scheme:

4 $$\begin{array}{c}{\rm{miniminze}}{}_{f,u}{\rm{\Phi }}({T}_{u}\cdot f)\\ s.t.{\Vert Af-y\Vert }^{2} < \delta \end{array}$$

where *f* represents the estimated dynamic images, *A* is the CBCT imaging system matrix with elements of *a*_*ij*_, *y* denotes the projection measurements. Operator Φ is the cube based sparsity penalties, while ${T}_{u}\cdot f$ denotes the dataset that includes a sequence of 4D matrix of the tracked cubes. Operator *T*_*u*_ includes two steps: (1) Track three-dimensional blocks with the motion vector, and (2) Rearrange the three-dimensional blocks into 4D matrix.

To optimize the problem in (4), in this work, inspired by the techniques used by Pan *et al*.^46^, we present an efficient way to solve (4), which can be summarized as:

(1) SART^47^ step:

5 $${f}_{j}^{(n+1/2)}={f}_{j}^{(n)}+\lambda (\sum _{i}[{a}_{ij}\frac{{y}_{i}-\sum _{n=1}^{N}{a}_{in}\,{f}_{j}^{(n)}}{\sum _{n=1}^{N}{a}_{in}}]/\sum _{i}{a}_{ij})$$

Here *λ* is an over-relaxed factor.

(2) MgSS step:${f}^{(n+1)}=W({\tilde{{\rm{\Theta }}}}_{MgSS}^{\ast }({f}^{(n+1/2)}))$ Here, ${\tilde{{\rm{\Theta }}}}_{MgSS}^{\ast }({f}^{(n+1/2)})$ denotes the restored block-based clusters based on ${f}^{(n+1/2)}$ by using the HOSVD approach

6 $${{\rm{\Theta }}}_{MgSS}^{\ast }={\hat{S}}_{MgSS}{\times }_{1}{U}^{(1)}{\times }_{2}{U}^{(2)}{\times }_{3}{U}^{(3)}{\times }_{4}{U}^{(4)}$$

*W* is a weighted averaging operator indicates how the cubes are merged back into the image. The elements of *W* is the reciprocal of the times which one pixel was overlapped by different cubes.

In our implementation, the MgSS step is carried out after 10^th^ iteration of SART step until ${|{f}^{(n)}-{f}^{(n-1)}|}^{2} < \xi$ or reached the predetermined iterative number. Also one implicit motion vector field estimation step was performed using the intermediate reconstructions of SART.

## Results

### Data acquisition of digital simulation

#### NCAT phantom based simulation

In this work, the 4D NURBS-based Cardiac-Torso (NCAT)^48^ phantom, which is capable of providing the realistic model of human anatomy and simulating cardiac and respiratory motion simultaneously, was used for data simulation. A dynamic phantom with ten respiratory phases and breathing period set to be 5 s was generated. The maximum diaphragm motion and the maximum chest anterior–posterior motion is 20 mm and 5 mm. We manually added several tumors with different sizes, contrast and shapes in the right lung field of phantom to test the robustness of the MgSS algorithm. The diameters of the spherical tumors were: 6 mm, 10 mm, 16 mm and 22 mm. The long diameter and short diameter of the non-spherical tumor is: 28 mm and 22 mm. For all simulations, the size of the digital phantom is 256 × 256 × 150, with voxel size of 2 × 2 × 2 mm^3^. The projections were generated by utilizing fast ray-tracing technique. The projection size of each angular view is 300 × 200 with detector pixel size 2 × 2 mm^2^. In the process of simulation, projection views are evenly distributed over 360 degrees with the projection number of each phase range from 21 to 51. The noisy signal *S*_*i*_ at each detector bin *i* was simulated based on the Poisson noise model:

7 $${S}_{i}=Poisson({I}_{0}\,\exp (-{y}_{i}))+Normal(0,{\sigma }_{e}^{2})$$

Here *I*_0_ and ${\sigma }_{e}^{2}$ represent the incident x-ray intensity and the background noise, respectively. *I*_0_ is set to be 2 × 10^6^ and ${\sigma }_{e}^{2}$ is chosen to be 10.

#### 4DCT based simulation

To further evaluate the performance of the MgSS algorithm, the 4DCT based digital phantom simulation was also performed. The 4DCT images were acquired on a 16-slice helical CT scanner (Brilliance Big Bore, Philips Medical Systems, Andover, MA). The three dimensional CT volumes at each phase were first interpolated to be isotropic data set with voxel size 1.0254 × 1.0254 × 1.0254 mm^3^. Then CB projections were computed from the reference 3D CT image using the projection matrix. The scan geometry was chosen according to the Varian On-Board Imager® and True Beam™ CBCT units.

### Data acquisition of patient

The patient data was downloaded from an open data website (http://wiki.openrtk.org/index.php/ RTK/Examples/MCCBCTReconstruction). The cone-beam projections were acquired on the Elekta Synergy system. The clinical dataset consisted of 644 projections and were sorted to 10 phases based on the AS method^49^. The size of the digital flat panel is 512 × 512 with the pixel size of 0.8 × 0.8 mm^2^. In our experiments, the isotropic reconstruction target resolution was set to be 1.2 × 1.2 × 1.2 mm^3^ on a 256 × 256 × 200 matrix.

### Evaluation metrics

To quantitative evaluate the performance of the proposed algorithm, we calculate the relative root mean square error (rRMSE) between the phantom images and the reconstructions. The rRMSE is defined as:

8 $${\rm{rRMSE}}=\sqrt{\sum _{m=1}^{Q}{|{f}_{m}-{f}_{{\rm{p}},m}|}^{2}/\sum _{m=1}^{Q}{|{f}_{{\rm{p}},m}|}^{2}}$$

Here, *f* denotes the target image, *f*_*p*_ denotes the phantom image, and *m* is the voxel index.

The universal quality index^50^ (UQI) index was utilized to conduct region of interest (ROI) based analysis by evaluating the degree of similarity between the reconstructed and the reference images. We select ROIs including the tumor and lung details within the reconstructed and reference images, the mean, variance and covariance of intensities in the ROIs can be respectively calculated as:

9 $$\bar{f}=\frac{1}{Q}\sum _{m=1}^{Q}{f}_{m},\quad \,\,{\sigma }^{2}=\frac{1}{Q-1}\sum _{m=1}^{Q}{({f}_{m}-\bar{f})}^{2}$$

10 $${\bar{f}}_{ture}=\frac{1}{Q}\sum _{m=1}^{Q}{f}_{ture,m},{\sigma }_{ture}^{2}=\frac{1}{Q-1}\sum _{m=1}^{Q}{({f}_{ture,m}-{\bar{f}}_{ture})}^{2}$$

11 $$cov(f,{f}_{ture})=\frac{1}{Q-1}\sum _{m=1}^{Q}({f}_{m}-\bar{f})({f}_{ture,m}-{\bar{f}}_{ture})$$

the *f*_*ture*_ denotes the golden standard image, *m* is the voxel index, and *Q* denotes the number of voxels within the ROI. The UQI can be calculated as follows:

12 $$UQI=\frac{2cov(f,{f}_{ture})}{{\sigma }^{2}+{\sigma }_{ture}^{2}}\frac{2{\bar{f}}_{ture}\bar{f}}{{\bar{f}}^{2}+{\bar{f}}_{ture}^{2}}$$

UQI measures the intensity similarity between the two images, and its value ranges from zero to one. A UQI value closer to one suggests better similarity to the reference image.

### Digital NCAT phantom study

#### Visual inspection

**Figure 2:**
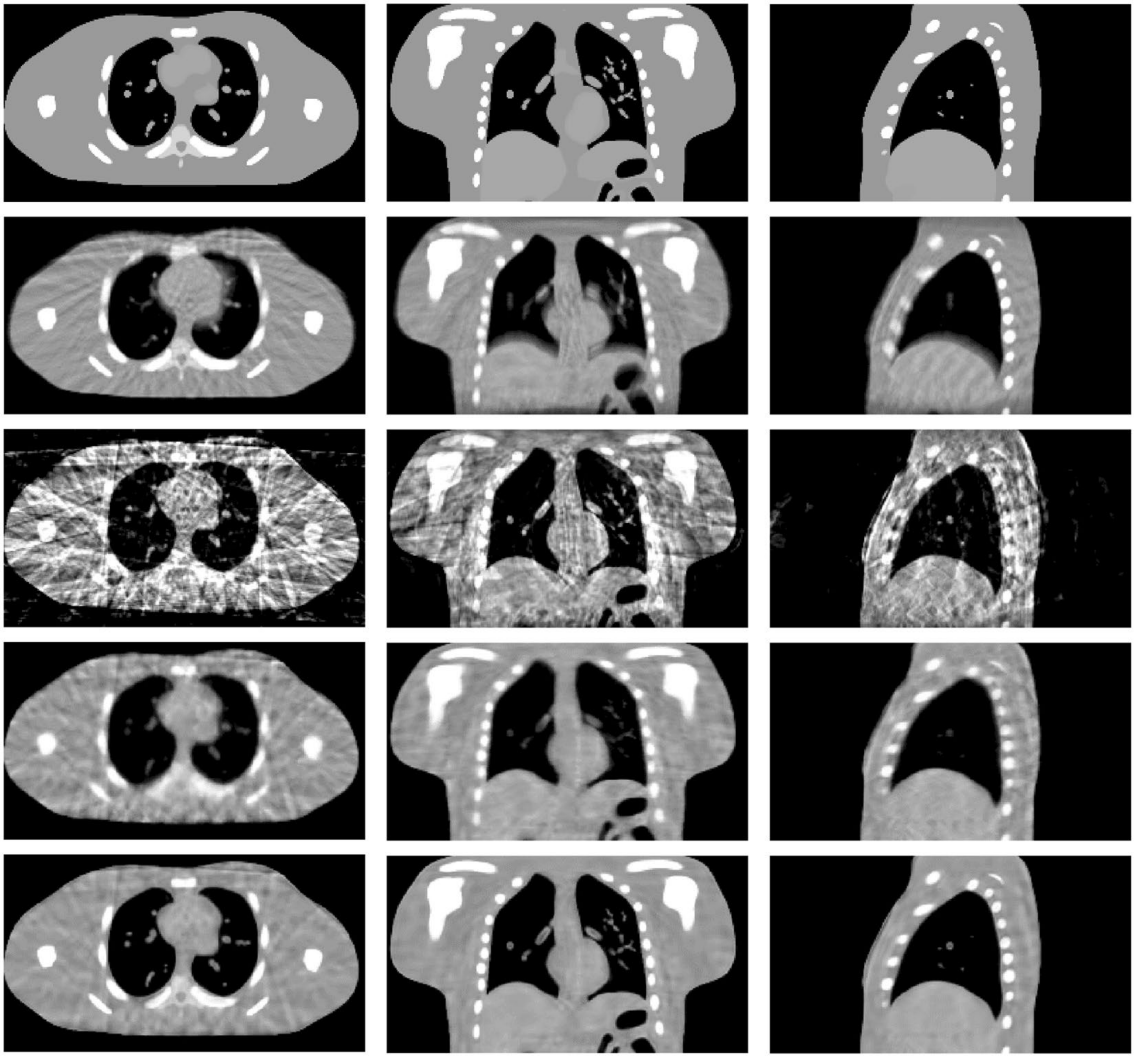
Results of 4D NCAT phantom with 21 projections for each phase. First row shows the begin-expiration phase of digital phantom. Second row shows the 3D-CBCT reconstructed from all projections by FDK. The third to last rows show 4D-CBCT images at the begin-expiration phase reconstructed by using FDK, SART-TV and proposed MgSS algorithm, respectively. The transverse, coronal, and sagittal planes have been shown in the first, second and third columns, respectively.

**Figure 3:**
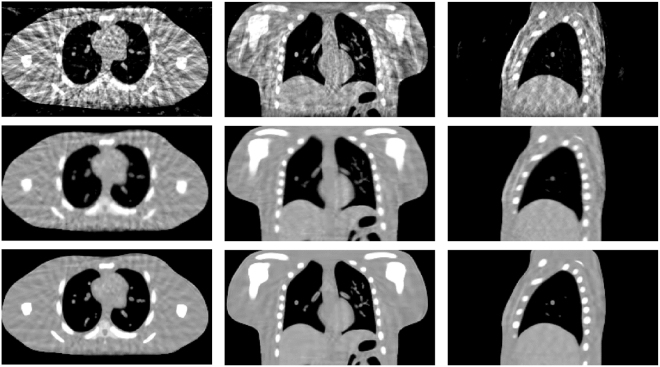
Reconstructions of 4D NCAT phantom with 31 projections for each phase. The first to third rows show 4D-CBCT at the begin-expiration phase reconstructed by FDK, SART-TV and proposed MgSS algorithms, respectively.

**Figure 4:**
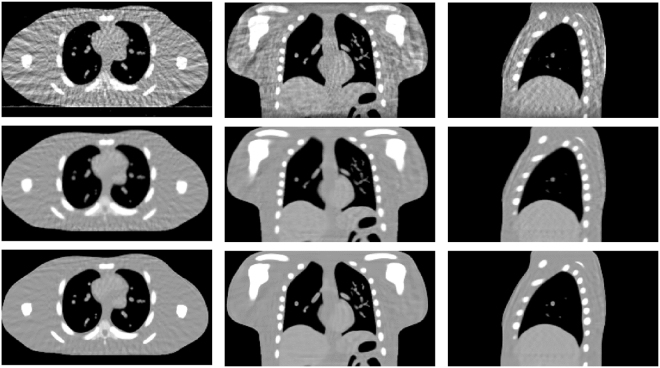
Reconstructions of 4D NCAT phantom with 51 projections for each phase. The first to third rows show 4D-CBCT at the begin-expiration phase reconstructed by FDK, SART-TV and proposed MgSS algorithms, respectively.

Figure 2 shows the results of the NCAT phantom reconstructed by using different methods at transverse, coronal, and sagittal planes for phase #1 with the projection number set to be 21. The columns one to three show the transverse, coronal, and sagittal images, respectively. First row in Fig. 2 shows the designed digital phantom images. The second row shows the results which were reconstructed by using the FDK algorithm from all projections. As we can see, the motion blurring artifacts are obvious. The third row shows the phase correlated 4D-CBCT images reconstructed by the FDK algorithm, and we can see that the FDK reconstructions are full of noise and streak artifacts. For the SART-TV reconstructions, the fine structures inside the lung area are severely blurred though the view aliasing artifacts are suppressed. Compared to FDK and SART-TV algorithms, the proposed MgSS approach can yield images with superior quality. Moreover, Figs 3 and 4 demonstrate the reconstructions of -31 and -51 views, which further illustrate the gains of the proposed MgSS approach.

#### Quantitative evaluation

Table 1 presents the results of rRMSE measures with the projection views range from 21 to 51 for each phase. The rRMSEs of ten phases reconstructions by using the proposed MgSS reduced by an average of 43% compared to those of FDK reconstructions. It can be obviously seen that the MgSS algorithm can achieve smaller rRMSE values compared to the other algorithms, which suggests the promising performance of the proposed MgSS approach.

**Table 1:** rRMSE measures on the reconstructions of the FDK, SART-TV and MgSS reconstructions, respectively.

Views	Method	PhaseNumber (Tumor diameter:10 mm)									
		one	two	three	four	five	six	seven	eight	nine	ten
21	FDK	8.72E-03	8.77E-03	8.90E-03	9.02E-03	9.11E-03	9.14E-03	9.13E-03	9.04E-03	8.90E-03	8.72E-03
	SART-TV	2.16E-03	2.16E-03	2.17E-03	2.15E-03	2.16E-03	2.17E-03	2.17E-03	2.16E-03	2.18E-03	2.19E-03
	MgSS	9.11E-04	9.10E-04	9.09E-04	9.05E-04	9.07E-04	9.10E-04	9.09E-04	9.09E-04	9.15E-04	9.17E-04
31	FDK	8.33E-03	8.38E-03	8.53E-03	8.67E-03	8.77E-03	8.83E-03	8.82E-03	8.74E-03	8.59E-03	8.41E-03
	SART-TV	1.99E-03	1.93E-03	1.97E-03	1.98E-03	1.96E-03	1.98E-03	1.97E-03	1.98E-03	1.97E-03	1.98E-03
	MgSS	8.73E-04	8.74E-04	8.74E-04	8.70E-04	8.71E-04	8.71E-04	8.68E-04	8.67E-04	8.66E-04	8.69E-04
41	FDK	8.12E-03	8.07E-03	7.92E-03	7.79E-03	7.68E-03	7.62E-03	7.63E-03	7.71E-03	7.86E-03	8.04E-03
	SART-TV	1.80E-03	1.81E-03	1.82E-03	1.81E-03	1.83E-03	1.80E-03	1.79E-03	1.82E-03	1.81E-03	1.80E-03
	MgSS	8.11E-04	8.10E-04	8.11E-04	8.08E-04	8.11E-04	8.10E-04	8.10E-04	8.09E-04	8.11E-04	8.13E-04
51	FDK	8.00E-03	7.96E-03	7.82E-03	7.68E-03	7.58E-03	7.52E-03	7.53E-03	7.62E-03	7.76E-03	7.93E-03
	SART-TV	1.74E-03	1.75E-03	1.74E-03	1.73E-03	1.76E-03	1.74E-03	1.75E-03	1.76E-03	1.73E-03	1.74E-03
	MgSS	7.96E-04	7.93E-04	7.96E-04	7.92E-04	7.94E-04	7.93E-04	7.92E-04	7.89E-04	7.93E-04	7.95E-04

**Figure 5:**
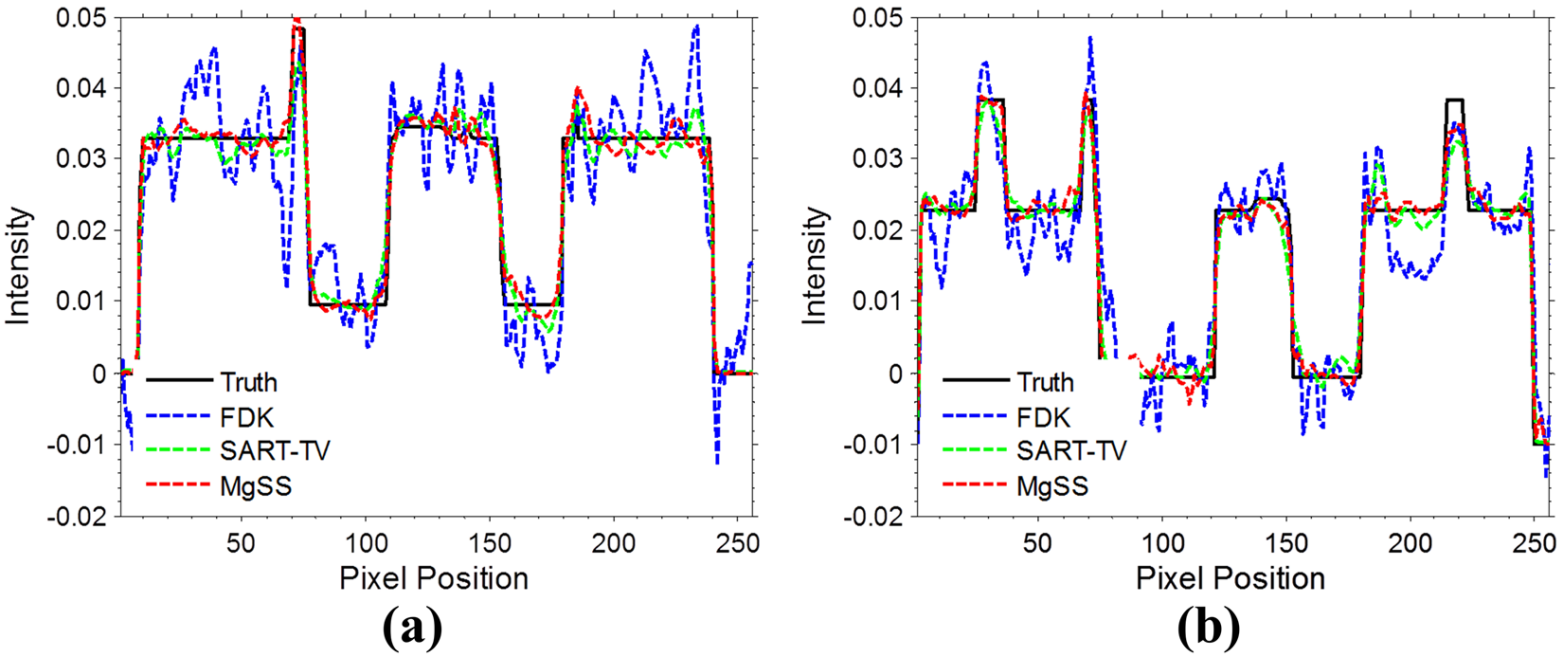
Horizontal profiles of reconstructions shown in Fig. 2. The profiles located at the pixel position x from 0 to 256 and y = 46 (**a**), y = 67 (**b**). The ‘black line’ generated from the NCAT phantom acts as the ground-truth for comparison.

Figure 5 illustrates the horizontal profiles of the transverse plane images in Fig. 2. It can be observed that the profiles obtained from the MgSS reconstructions match much better than the others, which suggest that the present MgSS approach achieves more noticeable gains than other approaches.

#### Motion trajectory accuracy

**Figure 6:**
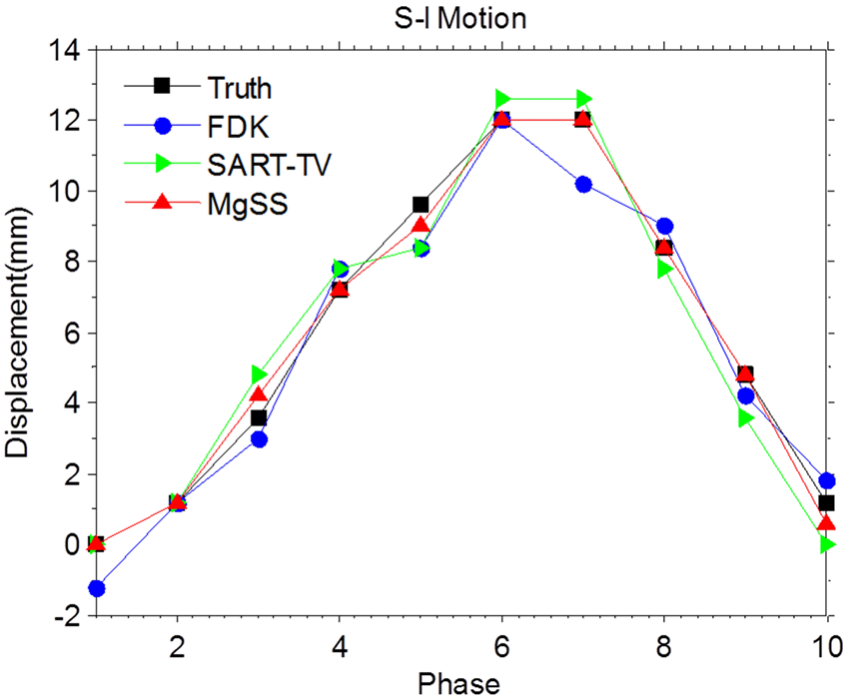
Motion trajectories extracted from the result of 4D-CBCT along the superior–inferior direction.

Because of the various applications of IGRT is interested in the tumor position information, we are devoted to extract the motion trajectories from different reconstruction schemes: FDK, SART-TV and the proposed MgSS algorithms. We define the motion trajectories which extracted from the NCAT phantom with high-contrast tumor as a reference. In this paper, we extract the movement information from the center of tumor. The motion trajectories have been showed in Fig. 6. As we can see, the motion information of tumor extracted from MgSS algorithm matches well with the reference trajectory, while the trajectories extracted from the FDK diverges the reference trajectory.

#### Low contrast lesion detection study

**Figure 7:**
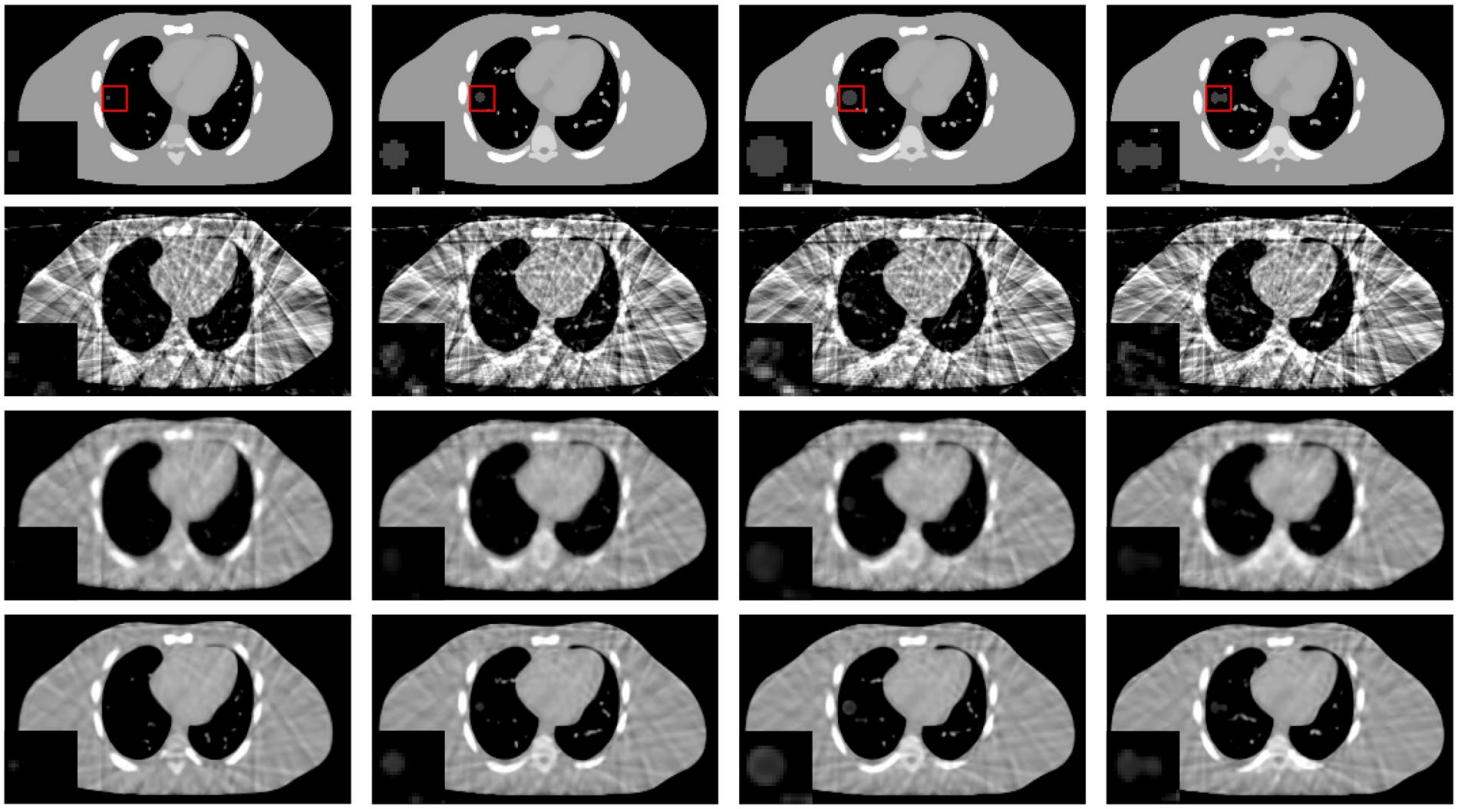
Reconstructions of 4D NCAT phantom with 21 projections. The first to the fourth columns show the transverse planes of 4D NCAT phantom with tumors of diameters: 6 mm, 16 mm, 22 mm and 28 mm, respectively.

**Figure 8:**
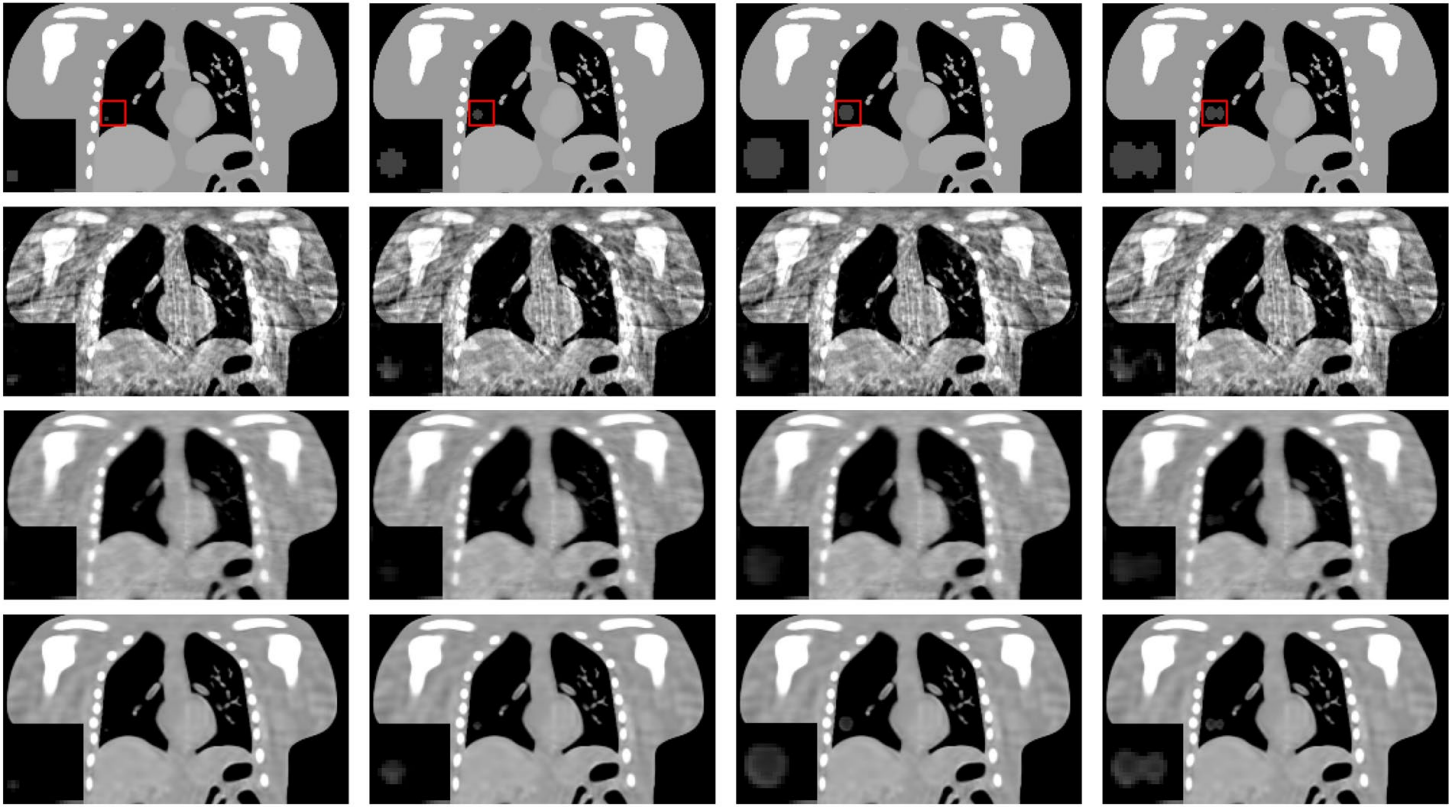
Reconstructions of 4D NCAT phantom with 21 projections. The first to the fourth columns show the coronal planes of 4D NCAT phantom with tumors of diameters: 6 mm, 16 mm, 22 mm and 28 mm, respectively.

**Figure 9:**
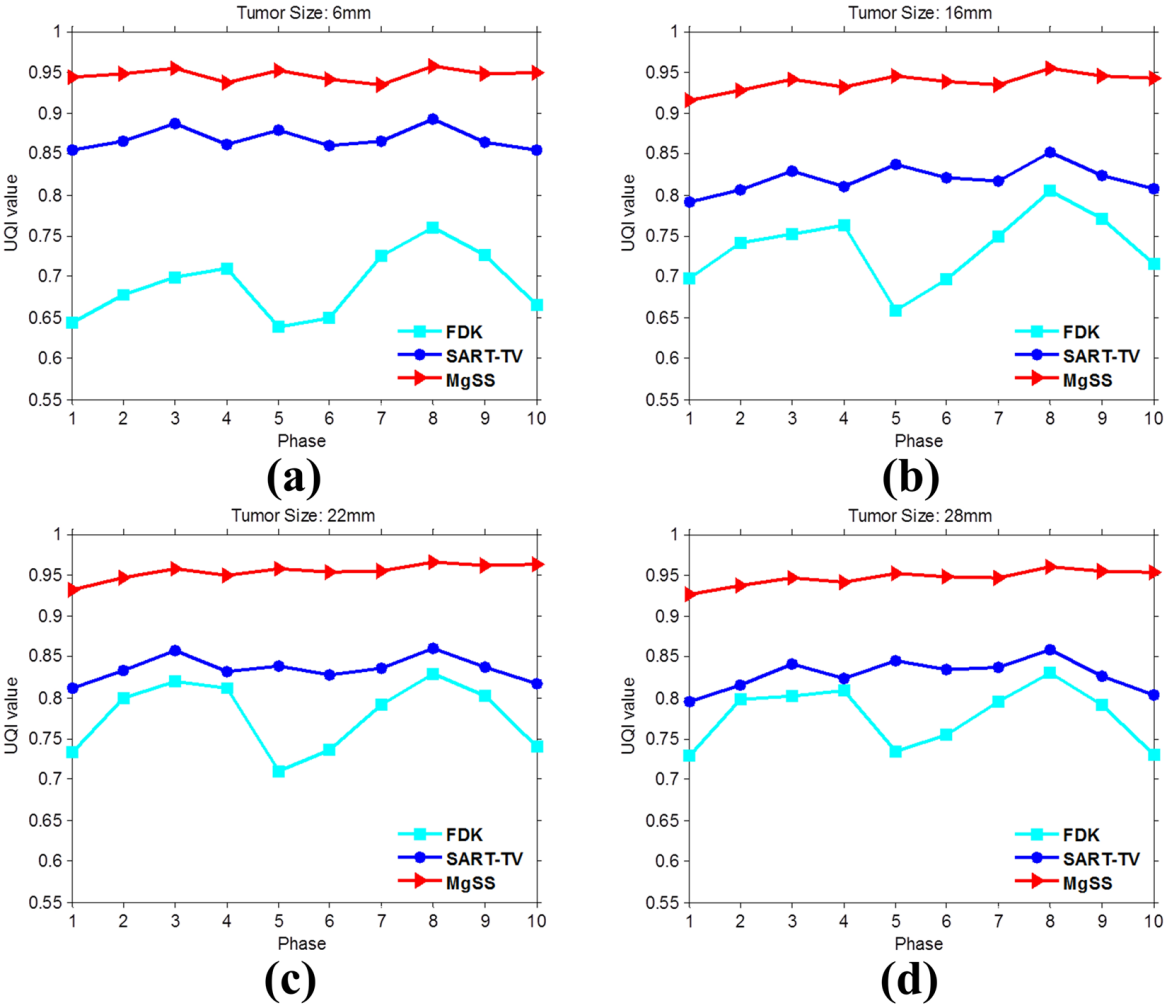
The UQI measures on the ROIs in Fig. 8 for ten phase.

To test the robustness of the MgSS algorithm in reproducing the low contrast lesion, Figs 7 and 8 present the reconstructions of NCAT phantom with different tumor sizes and shapes at the transverse and coronal planes. The first row shows the designed phantom image used for visual comparison. The second to fourth rows show 4D-CBCT at the begin-expiration phase reconstructed by using FDK, SART-TV and proposed MgSS algorithms, respectively. It can be seen, the low contrast tumors in the NCAT phantom can’t be completely rebuilt by the FDK and SART-TV algorithms. The morphology of tumors is partly destroyed. The phenomenon is particularly evident for tumors with small size. On the contrast, the MgSS algorithm can mostly recover the tumors with relatively complete morphological structures. Furthermore, Fig. 9 presents the UQI test on the ROIs shown in Fig. 8. All the results suggest that, with the introduction of phase-correlated information, the MgSS algorithm has better lesion detection ability than algorithms that only using the single phase information.

#### Parameter selection

In our method, there are two parameters need to be tuned: (1) the tracked cube size; (2) the soft-thresholding coefficients for the HOSVD processing. The cube size plays an important role in our work as it not only directly affects the accuracy of the results, but also affects the computation time. Traditionally, for the block-based techniques, the size of the cube is an empirical parameter specified by the user. A reasonable cube size can help us attend to the local structure characteristics while removing undesirable distortions.

**Figure 10:**
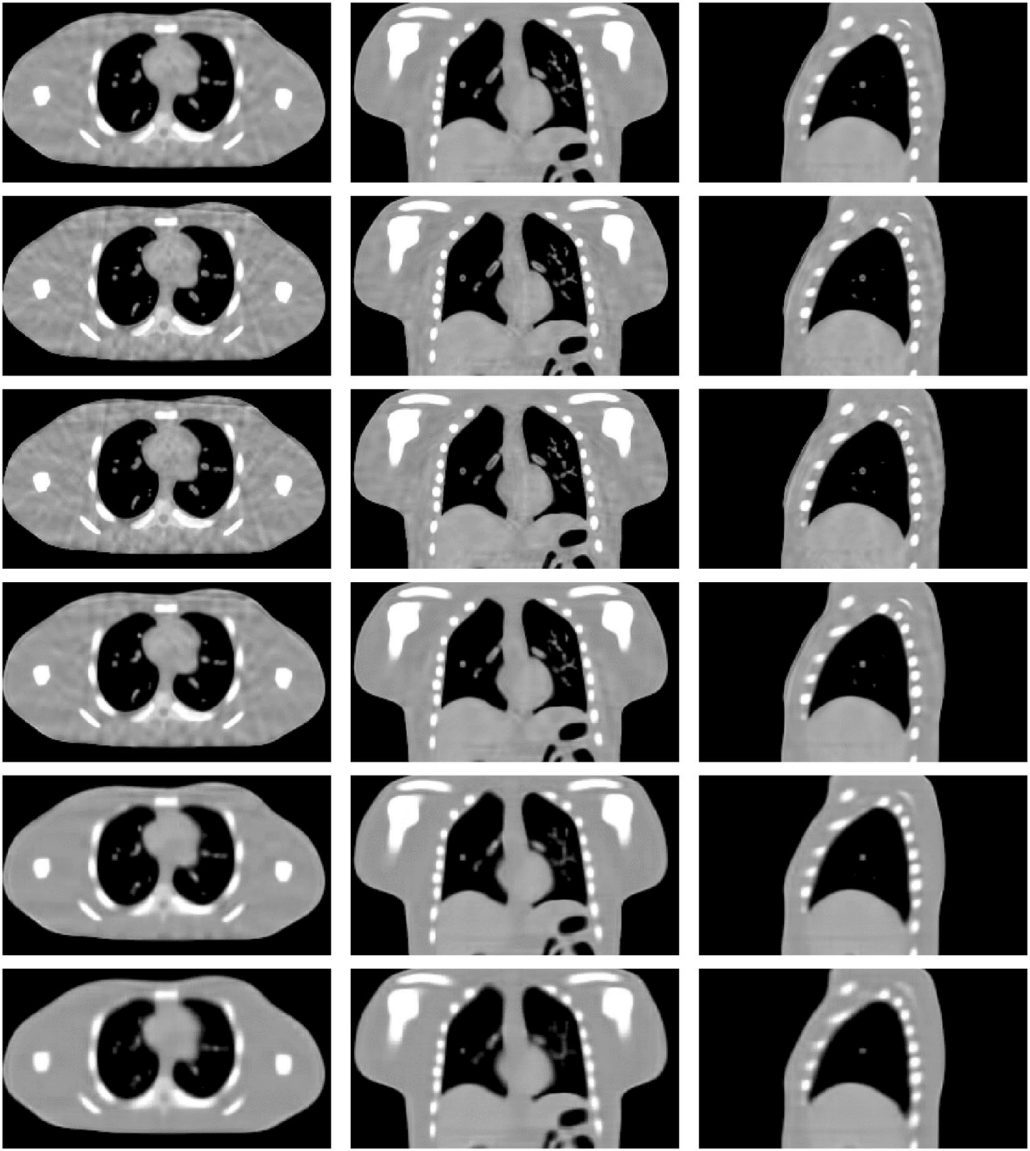
Ten phases reconstructions from -21 projections with block size set to be 5 × 5 × 5, 7 × 7 × 7, 9 × 9 × 9, 13 × 13 × 13, 17 × 17 × 17, 23 × 23 × 23.

**Figure 11:**
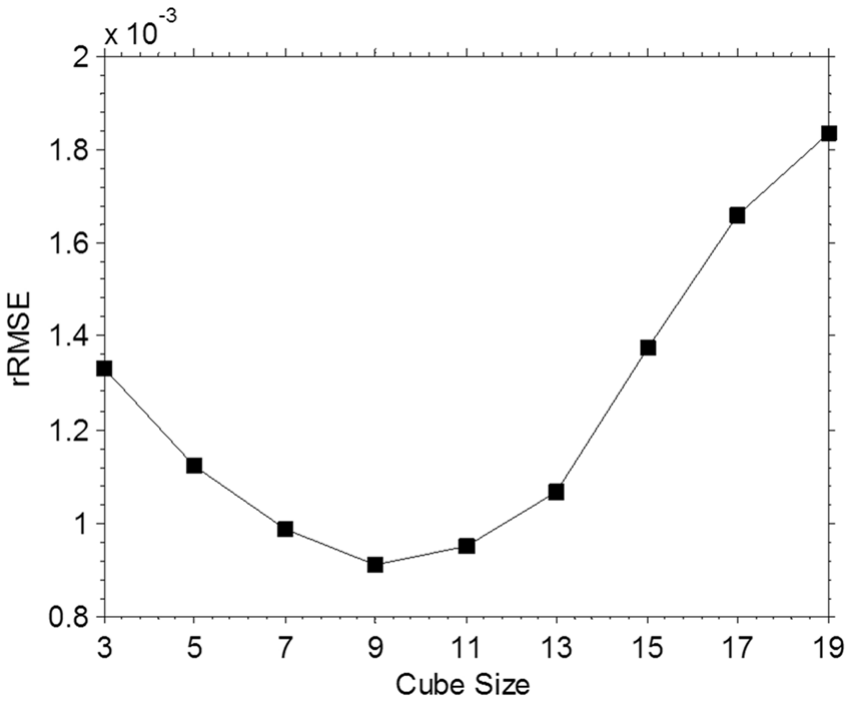
The averaged rRMSEs of ten phases reconstructions with different cube size.

To study the influence of the cube size on the proposed algorithm, we experimentally change the size of cube to get the associated reconstructions and calculate the rRMSEs between the reconstructions and the designed phantom image. We reconstruct the NCAT phantom from -21 projections with block size set from 3 × 3 × 3 to 23 × 23 × 23. Figure 10 shows the reconstructions of cube size to be 5 × 5 × 5, 7 × 7 × 7, 9 × 9 × 9, 13 × 13 × 13, 17 × 17 × 17, 23 × 23 × 23. As shown in Fig. 10, on one hand, boundary distortion could be observed when the cube size is too small. On the other hand, the image blur increases with the increase of the cube size. This phenomenon is due to the large cube contains too much structural information, the movement of central voxel in the cube is not enough to describe the whole cube. Thus, to generate high quality 4D-CBCT image, appropriate size of the cube is needed. Moreover, as shown in Fig. 11, the averaged rRMSEs of ten phases reconstructions indicate that with the cube size range from 7 × 7 × 7 to 11 × 11 × 11, we can get relative smaller rRMSEs. In order to balance the reconstruction quality and computational time, in our other experiments, the cube size was fixed to 9 × 9 × 9.

For the parameter of thresholding coefficients for the HOSVD processing, we choose with a threshold of $\sigma \sqrt{2\,\mathrm{log}\,{p}^{2}}$ to manipulate the coefficients of core tensor. This is also an experiential selection as used in other works^51^. Here, *σ* is defined as the standard deviation of a uniform region in the intermediary images during the iteration and *p* is the cube size.

#### Algorithm convergence

**Figure 12:**
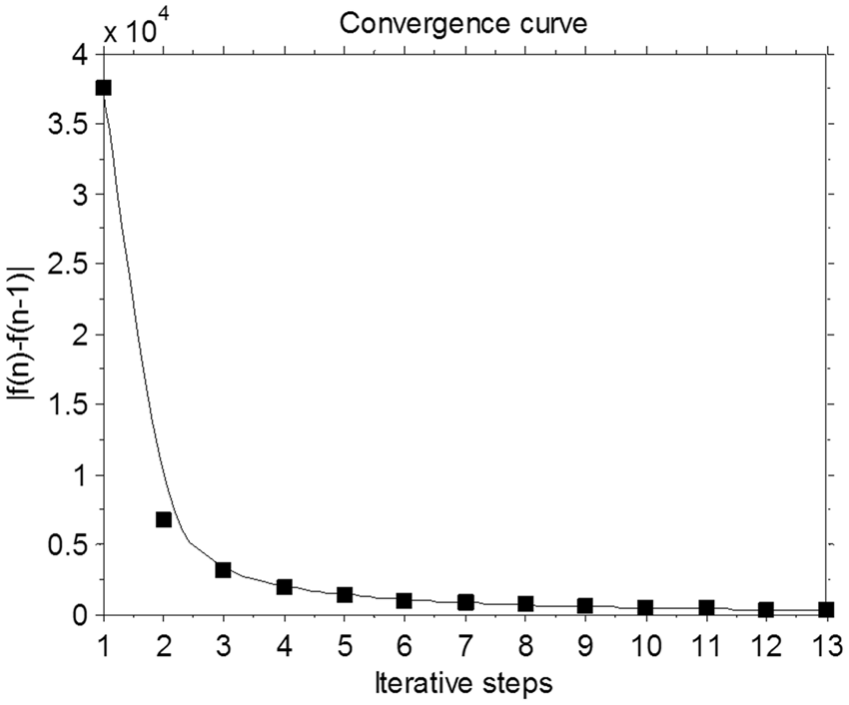
The convergence curve of the MgSS algorithm.

To validate and analyze the convergence of the present MgSS method, the $|{f}({\bf{n}})-{f}({\bf{n}}-1)|$ (absolute value of differences between two adjacent estimations) measuring on the entire to-be-reconstructed NCAT phantom image were performed. Figure 12 shows the $|{f}({\bf{n}})-{f}({\bf{n}}{-}1)|$ measures with respect to the number of iterations. Results show that the present MgSS algorithm can yield a steadily convergence solution.

#### Influence of motion tracking

**Figure 13:**
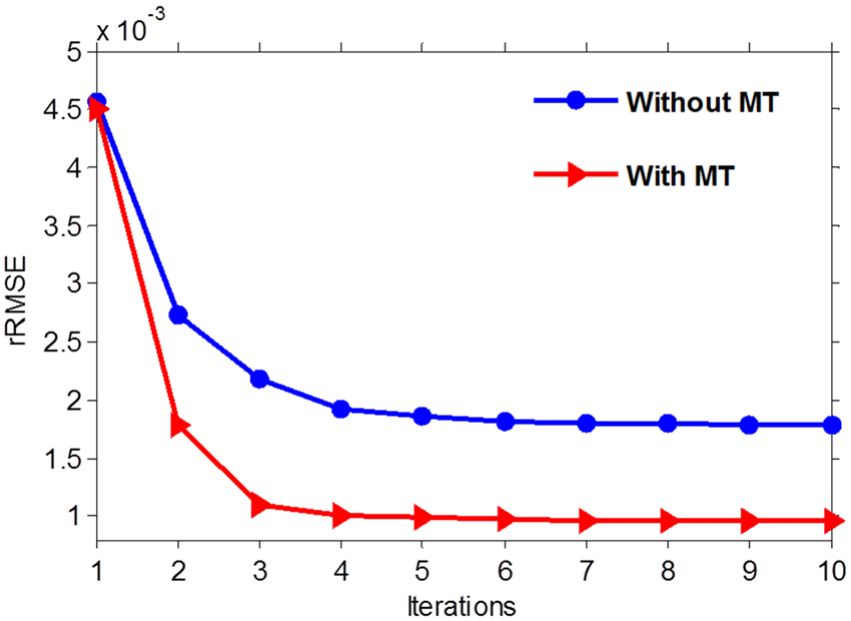
The rRMSE measures as a function of the number of iterations for the MgSS algorithm with and without motion tracking.

To demonstrate the effect of the estimated motion fields on the reconstruction, we plot the rRMSE measures as a function of the number of iterations for the MgSS with and without motion tracking. Figure 13 shows the benefits of motion guidance, as MgSS with motion tracking can obtain reduced rRMSE as compared to MgSS without motion tracking. Figure 13 also illustrates the convergence of the proposed MgSS algorithm.

### Realistic 4D CT based digital phantom study

**Figure 14:**
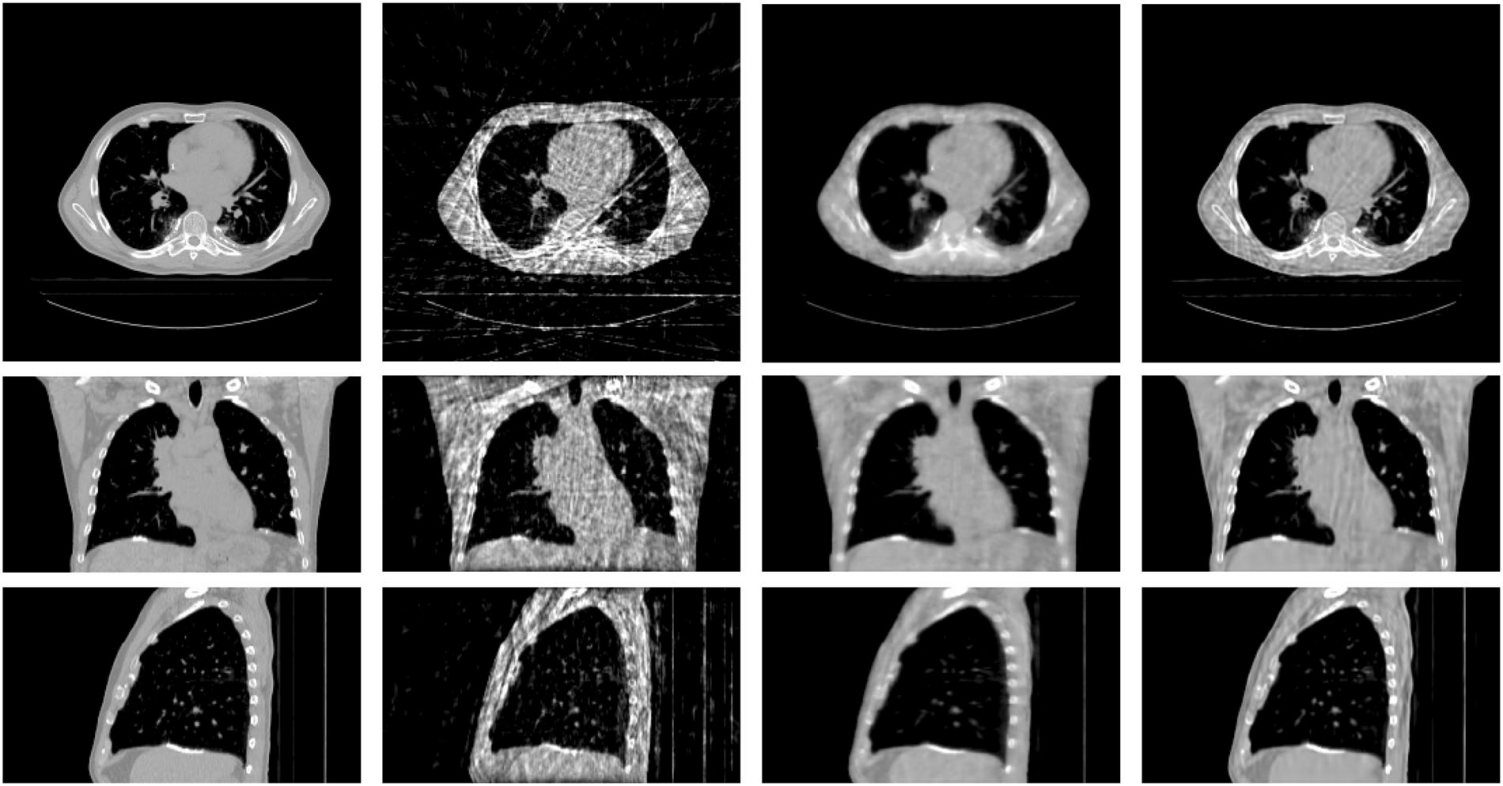
Results of realistic digital phantom with 21 projections for each phase. First column shows the begin-expiration phase of the 4DCT images. The second to last columns show 4D-CBCT images at the begin-expiration phase reconstructed by using FDK, SART-TV and proposed MgSS algorithms, respectively.

**Figure 15:**
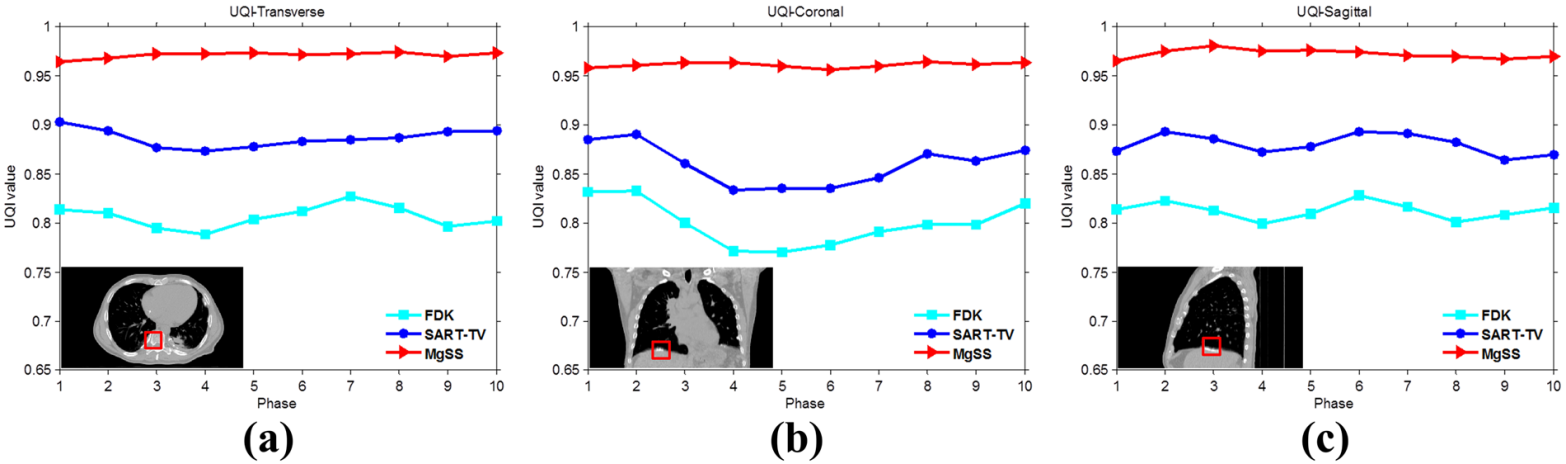
The UQI tests on ten phases reconstructions from -21views projection at the transverse, sagittal and coronal planes separately.

Figure 14 shows the results of the realistic digital phantom reconstructed by using different methods at transverse, coronal, and sagittal planes for phase #1 with the projection number set to be 21. First column in Fig. 14 shows the 4DCT images using as the golden standard for comparison. The second column shows 4D-CBCT images reconstructed by the FDK algorithm. It can be seen that the FDK reconstructions are seriously contaminated by noise and artifacts, and some anatomical structures can’t be clearly seen. For the SART-TV reconstruction, some fine structures have been erased though most of the view aliasing artifacts are suppressed. Compared to FDK and SART-TV algorithms, the proposed MgSS approach can yield images with superior quality. In addition, the UQI test between the reconstructions and golden standard image were calculated. Figure 15 shows the test results of then phases at the transverse, sagittal and coronal planes, separately. For then phases, the UQI values of MgSS reconstructions are always higher than 0.95, which suggests the promising performance of the proposed MgSS algorithm.

### Patient study

**Figure 16:**
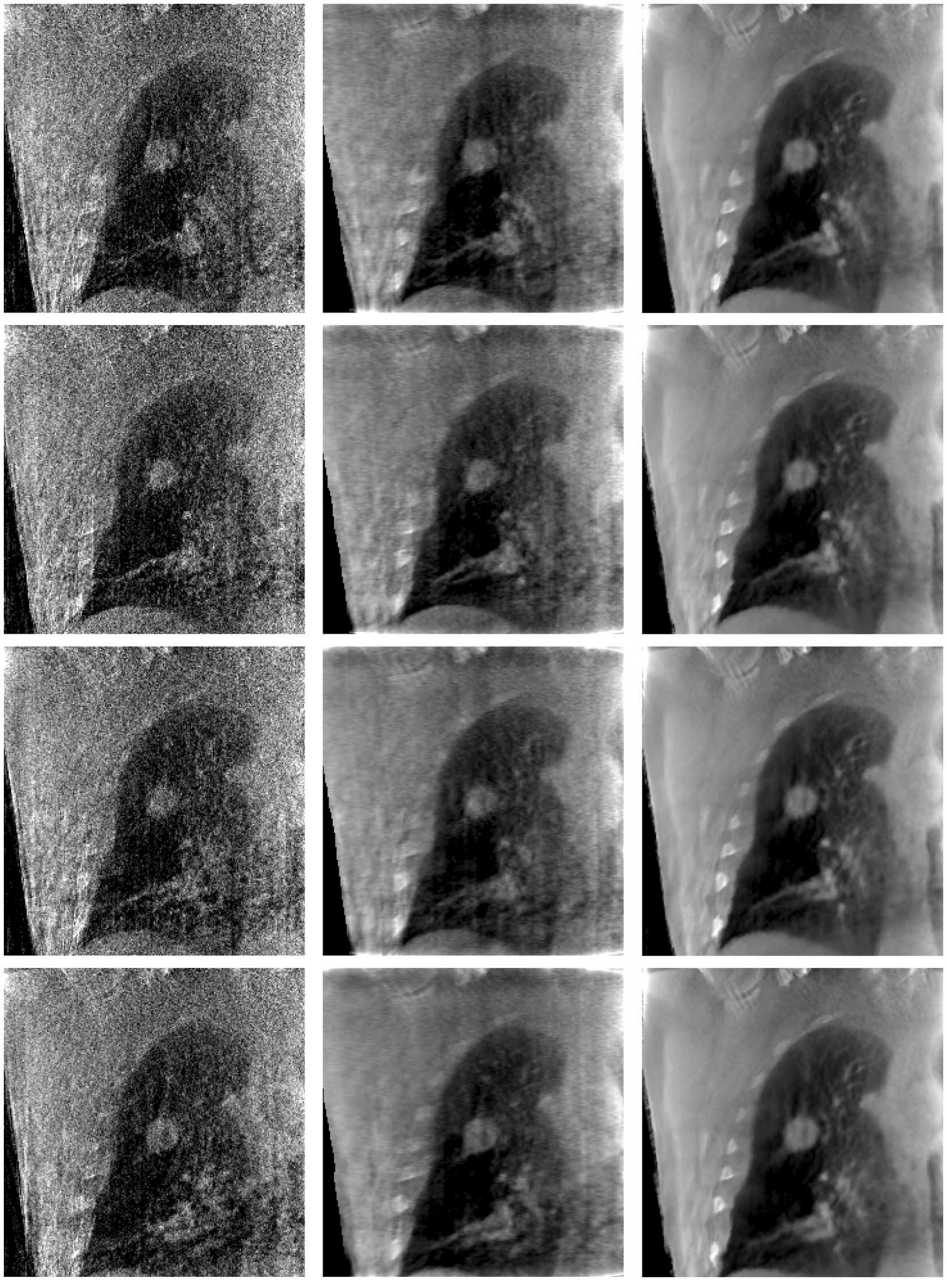
Results of patient data reconstructed by using different methods. The left to the right columns show the 4D-CBCT reconstructed by FDK, SART-TV and proposed MgSS algorithm, respectively.Each row shows images at the different phases in the breathing cycle: 20–30%, 40–50%, 60–70%, 80–90%.

**Figure 17:**
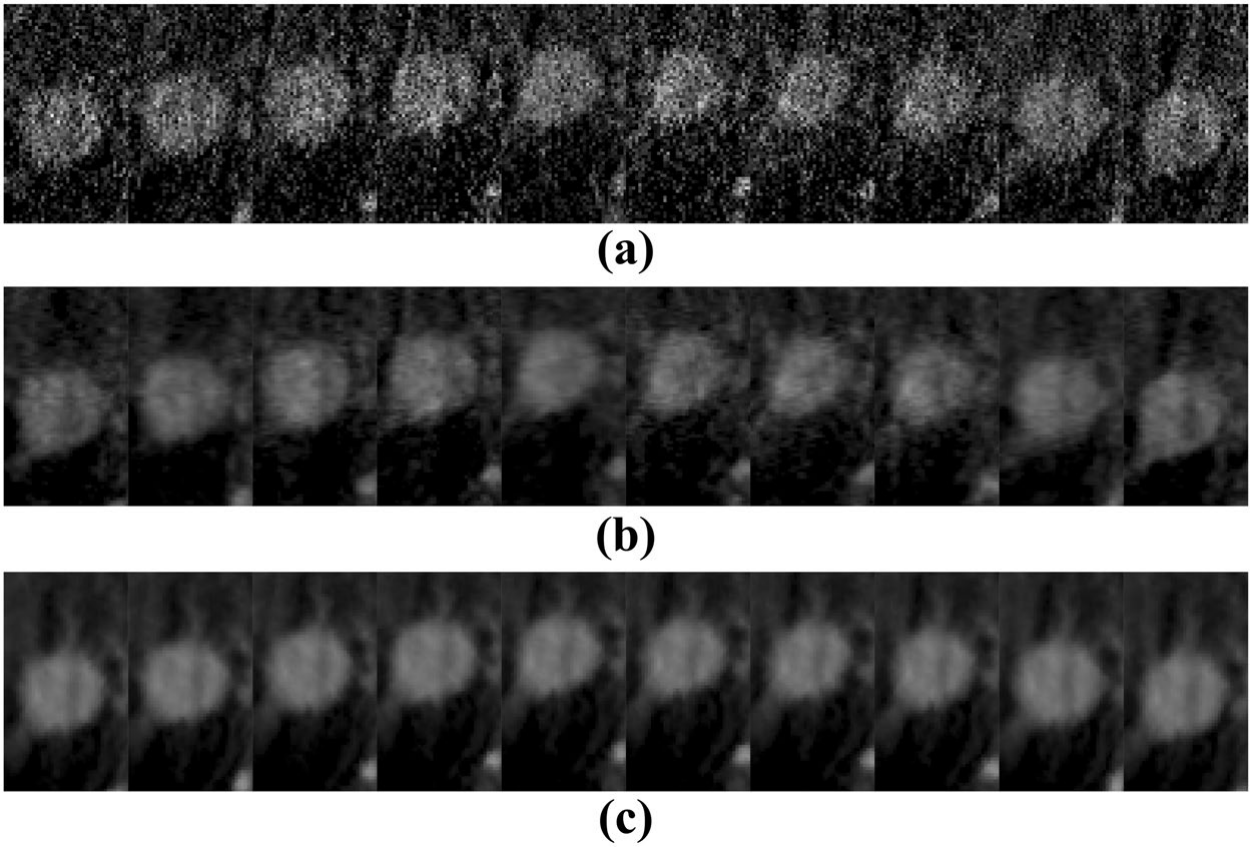
The zoomed tumor areas in the reconstructions of ten phases. The first to the third rows show the tumor image which were reconstructed by FDK, SART-TV, and MgSS, respectively.

Figure 16 shows the representative reconstructions of patient data by using the FDK, SART-TV and MgSS methods. Each row of Fig. 16 shows the image of 4D-CBCT at different phase in respiratory cycle: 20–30%, 40–50%, 60–70%, 80–90%. The first column in Fig. 16 shows the sagittal view of 4D-CBCT images reconstructed by conventional FDK. Because of the limited number of projections at each phase, severe noise and artifacts present in the FDK reconstruction. The second column of Fig. 16 shows the results of SART-TV algorithm. Although noise and artifacts have been remarkably reduced, details within the lung area and edges of bony structure can’t be clearly seen. Last column of Fig. 16 shows the images reconstructed by MgSS method, from which we can observe noise is suppressed. The boundaries of bony structures as well as fine structures inside the lung are well preserved. To further illustrate the performance of our algorithm, zoomed images of the tumor areas in the then phasesof the reconstructions by using different reconstruction are presented (Fig. 17).

## Discussion

In this work, we developed a MgSS algorithm to improve the image quality of 4D-CBCT. This algorithm is developed on the assumption that there exists high structural similarity between the images of neighbored phases, which is similar with Chen’s work on dynamic MR reconstruction^43^. In Chen’s work, two dimensional patches are tracked with estimated motion maps and then SVD was used to decompose the tacked cluster. For the standard SVD, the image blocks are manually vectorized and then the image structural properties in the spatial domain are ignored. For the proposed MgSS algorithm, the 3D-MVFs between phases were utilized to track the three dimensional cubes and then form the cluster Θ_*MgSS*_ with the size of ${N}_{b}\times {N}_{b}\times {N}_{b}\times {N}_{t}$. To preserve the structural properties, in this work, the higher order singular value decomposition which is able to directly decompose dynamic datasets into a multidimensional singular matrix rather than unfolding the cubes into column vectors, was used to process the four dimensional cluster Θ_*MgSS*_. The MgSS algorithm can reveal the fine details shared by grouped blocks and preserve the essential unique features of each individual block. Results of digital phantom and patient data demonstrate the proposed approach can significantly suppress the view aliasing artifacts and noise.

One important step in the proposed MgSS algorithm is: cube based motion tracking. We utilize the Real-Time Image-based Tracker (RTIT)^36^ toolbox to obtain the voxel-by-voxel displacement maps between 3D images of different phases. Specifically, we rely mainly on the displacement of the center voxel of cube in the current phase to determine the cube in the next phase. We assume the displacements of region are changing smoothly. Thus, the central voxel is sufficient to describe the cube motion. And in this case, the accuracy of 3D-MVFs estimation would play an important role in finding cubes with similar structures. In this work, the initial 3D-MVFs are generated after the first SART iteration, which may not so accurate. Notably, as the iteration goes on, the 3D-MVFs would be updated with intermediate reconstructions, and therefore both the image quality and the accuracy of the 3D-MVFs will be improved. Other approaches such like the 3D-MVFs initialized with those got from the 4D planning CT of the same patient can be considered. There is a very important parameter need to be tuned for the proposed MgSS approach: the tracked cube size. A large size cube may include more details and local geometry, but on the other side, if the cube size is too large, the central voxel will be insufficient to describe the whole cube motion and also result in increasing computation time. Thus, the proper selection of the cube size is critical. In our studies, we change the block size from 3 × 3 × 3 to 23 × 23 × 23 and generate the reconstructions as shown in Fig. 10 and rRMSEs between the reconstructions and the phantom images. For visual inspection, too small or large cube size would lead to image blur or distortion. The rRMSE results suggest that when the cube size set to be 9 × 9 × 9, we can get relative higher quality image with smaller rRMSE for the NCAT phantom. Also as illustrated in other studies of HOSVD technique, the appropriate selection of the cube size should base on the structural complexity of the target image and may be different for different images. But overall, in our work for lung CT imaging, we found the cube size set to be 9 × 9 × 9 is robust enough to balance the structural information and computation time. Another issue of the proposed MgSS algorithm is the heavy computation burden. It takes about 30 minutes on a desktop computer (3.60 GHz Intel(R) i7 CPU with 8GB RAM) to run one iteration. On one hand, as mentioned above, we can short the computation time with a relatively small cube size as well as an elegant initialization. On the other hand, we can use a step of N_step_ pixels in transverse, coronal, and sagittal directions, respectively. Hence, the number of overlapping cubes is decreased by 1/N_step_^3^. But with large N_step_ or large motion, gaps between moved blocks may appear, then a mask of the uncovered areas (gaps) on the n-th frame after block motion tracking can be detected and non-motion tracking is performed for the gap blocks to avoid potential additional gaps. Other techniques including using the graphics processing unit (GPU) can be also considered to short the computation time.

In summary, we have developed a MgSS algorithm to improve the image quality of 4D-CBCT. This method effectively utilizes the correlated information from other phases to reconstruct any particular phase of 4D-CBCT. By enforcing the regional spatiotemporal sparsity on the tracked cubes, noise and artifacts can be suppressed and the image quality of 4D-CBCT can be substantially improved.

## References

[1] Jaffray DA, Siewerdsen JH, Wong JW, Martinez AA (2002). Flat-panel cone-beam computed tomography for image-guided radiation therapy. International Journal of Radiation Oncology* Biology* Physics.

[2] Oldham M (2005). Cone-beam-CT guided radiation therapy: A model for on-line application. Radiotherapy and oncology.

[3] Pouliot J (2005). Low-dose megavoltage cone-beam CT for radiation therapy. International Journal of Radiation Oncology* Biology* Physics.

[4] Chen GT, Kung JH, Beaudette KP (2004). Artifacts in computed tomography scanning of moving objects. Semin Radiat Oncol.

[5] Sonke J, Zijp L, Remeijer P, van Herk M (2005). Respiratory correlated cone beam CT. Medical physics.

[6] Li T (2006). Four-dimensional cone-beam computed tomography using an on-board imager. Medical physics.

[7] Lu J (2007). Four-dimensional cone beam CT with adaptive gantry rotation and adaptive data sampling. Medical physics.

[8] Ford EC, Mageras GS, Yorke E, Ling CC (2003). Respiration-correlated spiral CT: a method of measuring respiratory-induced anatomic motion for radiation treatment planning. Medical physics.

[9] Low DA (2003). A method for the reconstruction of four-dimensional synchronized CT scans acquired during free breathing. Medical physics.

[10] Pan T, Lee T, Rietzel E, Chen GT (2004). 4D-CT imaging of a volume influenced by respiratory motion on multi-slice CT. Medical physics.

[11] Feldkamp LA, Davis LC, Kress JW (1984). Practical cone-beam algorithm. JOSA A.

[12] Li T, Koong A, Xing L (2007). Enhanced 4D cone-beam CT with inter-phase motion model. Medical physics.

[13] Li T, Xing L (2007). Optimizing 4D cone-beam CT acquisition protocol for external beam radiotherapy. International Journal of Radiation Oncology* Biology* Physics.

[14] Li T, Schreibmann E, Yang Y, Xing L (2006). Motion correction for improved target localization with on-board cone-beam computed tomography. Phys Med Biol.

[15] Zhang H (2014). Few-view cone-beam CT reconstruction with deformed prior image. Med Phys.

[16] Wang J, Li T, Xing L (2009). Iterative image reconstruction for CBCT using edge-preserving prior. Medical physics.

[17] Brock RS, Docef A, Murphy MJ (2010). Reconstruction of a cone-beam CT image via forward iterative projection matching. Medical physics.

[18] Jia X, Dong B, Lou Y, Jiang SB (2011). GPU-based iterative cone-beam CT reconstruction using tight frame regularization. Physics in medicine and biology.

[19] Sidky EY, Pan X (2008). Image reconstruction in circular cone-beam computed tomography by constrained, total-variation minimization. Physics in medicine and biology.

[20] Sidky EY (2009). Enhanced imaging of microcalcifications in digital breast tomosynthesis through improved image-reconstruction algorithms. Medical Physics.

[21] Kinnon GCM, Bates RHT (1981). Towards Imaging the Beating Heart Usefully with a Conventional CT Scanner. IEEE Transactions on Biomedical Engineering.

[22] Leng S (2008). Streaking artifacts reduction in four-dimensional cone-beam computed tomography. Medical Physics.

[23] Leng S (2008). High temporal resolution and streak-free four-dimensional cone-beam computed tomography. Physics in Medicine & Biology.

[24] Bergner F (2009). Autoadaptive phase-correlated (AAPC) reconstruction for 4D CBCT. Medical physics.

[25] Kida S, Masutani Y, Nakano M, Imae T, Haga A (2012). Improvement of 4D Cone-beam CT image quality via iterative reconstruction method. Ieice Technical Report.

[26] Bergner F (2010). An investigation of 4D cone-beam CT algorithms for slowly rotating scanners. Medical Physics.

[27] Haralick RM (1979). Statistical and structural approaches to texture. Proceedings of the IEEE.

[28] Lee JS (1980). Digital Image Enhancement and Noise Filtering by Use of Local Statistics. IEEE Transactions on Pattern Analysis & Machine Intelligence.

[29] Aharon M, Elad M, Bruckstein A (2006). K-SVD: An algorithm for designing overcomplete dictionaries for sparse representation. IEEE Transactions on Signal Processing.

[30] Buades A, Coll B, Morel JM (2005). A Review of Image Denoising Algorithms, with a New One. Siam Journal on Multiscale Modeling & Simulation.

[31] Dabov K, Foi A, Katkovnik V, Egiazarian K (2007). Image denoising by sparse 3-D transform-domain collaborative filtering. IEEE Transactions on Image Processing.

[32] Liu, C. & Freeman, W. T. A high-quality video denoising algorithm based on reliable motion estimation. *European Conference on Computer Vision* 706–719 (2010).

[33] Dwivedi, A. & Shrivastava, S. K. An efficient and fast patch reordering approach for image denoising without losing structural information. *International Conference on Computer Communications* 1–6 (2014).

[34] Parameswaran, S., Luo, E. & Nguyen, T. Q. Patch Matching for Image Denoising Using Neighborhood-based Collaborative Filtering. *IEEE Transactions on Circuits and Systems for Video Technology*, 1–1 (2016).

[35] Costantini R, Sbaiz L, Süsstrunk S (2008). Higher order SVD analysis for dynamic texture synthesis. IEEE Transactions on Image Processing A Publication of the IEEE Signal Processing Society.

[36] Ries M (2010). Real-time 3D target tracking in MRI guided focused ultrasound ablations in moving tissues. Magn Reson Med.

[37] Zachiu, C., Senneville, B. D. D., Moonen, C. & Ries, M. A framework for the correction of slow physiological drifts during MR-guided HIFU therapies: Proof of concept. *Medical Physics* **42** (2015). 10.1118/1.4922403 26133614

[38] Zachiu C, Papadakis N, Ries M, Moonen C, Denis DSB (2015). An improved optical flow tracking technique for real-time MR-guided beam therapies in moving organs. Physics in Medicine & Biology.

[39] Lingala SG, Hu Y, Dibella E, Jacob M (2011). Accelerated Dynamic MRI Exploiting Sparsity and Low-Rank Structure: k-t SLR. IEEE Transactions on Medical Imaging.

[40] Cai JF, Jia X, Gao H, Jiang SB (2014). Cine Cone Beam CT Reconstruction Using Low-Rank Matrix Factorization: Algorithm and a Proof-of-Principle Study. IEEE Transactions on Medical Imaging.

[41] Akcakaya M (2011). Low-dimensional-structure self-learning and thresholding: regularization beyond compressed sensing for MRI reconstruction. Magn Reson Med.

[42] Vitanis V (2011). High resolution three-dimensional cardiac perfusion imaging using compartment-based k-t principal component analysis. Magn Reson Med.

[43] Chen X, Salerno M, Yang Y, Epstein FH (2014). Motion‐compensated compressed sensing for dynamic contrast‐enhanced MRI using regional spatiotemporal sparsity and region tracking: Block low‐rank sparsity with motion‐guidance (BLOSM). Magnetic resonance in medicine.

[44] Lathauwer LD, Moor BD, Vandewalle J (2000). On the Best Rank-1 and Rank-(R 1, R 2, …, R N) Approximation of Higher-Order Tensors. Siam Journal on Matrix Analysis & Applications.

[45] Pan H, Huang TZ, Ma T (2016). Two-step group-based adaptive soft-thresholding algorithm for image denoising. Optik - International Journal for Light and Electron Optics.

[46] Jorgensen JS, Sidky EY, Pan X (2013). Quantifying admissible undersampling for sparsity-exploiting iterative image reconstruction in X-ray CT. IEEE Trans Med Imaging.

[47] Andersen AH, Kak AC (1984). Simultaneous algebraic reconstruction technique (SART): a superior implementation of the ART algorithm. Ultrasonic imaging.

[48] Segars, W. P. *et al*. Development and application of the new dynamic Nurbs-based Cardiac-Torso (NCAT) phantom (2001).

[49] Zijp, L., Sonke, J. J. & Herk, M. V. Extraction of the Respiratory Signal from Sequential Thorax Cone-Beam X-Ray Images. In ICCR (2004).

[50] Wang Z, Bovik AC, Sheikh HR, Simoncelli EP (2004). Image quality assessment: from error visibility to structural similarity. IEEE Transactions on Image Processing A Publication of the IEEE Signal Processing Society.

[51] Dabov K, Foi A, Egiazarian K (2006). Image denoising with block-matching and 3D filtering. Proceedings of SPIE - The International Society for Optical Engineering.

